# TSPAN1: A Novel Protein Involved in Head and Neck Squamous Cell Carcinoma Chemoresistance

**DOI:** 10.3390/cancers12113269

**Published:** 2020-11-05

**Authors:** Yoelsis Garcia-Mayea, Cristina Mir, Laia Carballo, Josep Castellvi, Jordi Temprana-Salvador, Juan Lorente, Sergi Benavente, Juana M. García-Pedrero, Eva Allonca, Juan P. Rodrigo, Matilde E. LLeonart

**Affiliations:** 1 Biomedical Research in Cancer Stem Cells, Vall d’Hebron Research Institute (VHIR), Autonomous University of Barcelona, Passeig Vall d’Hebron 119-129, 08035 Barcelona, Spain; yoelsis.garcia@vhir.org (Y.G.-M.); cristina.mir@vhir.org (C.M.); laia.carballo@alumni.vhir.org (L.C.); joscastellvi@vhebron.net (J.C.); jtemprana@vhebron.net (J.T.-S.); 2 Genetic, Microbiology and Statistics Department, Faculty of Biology, University of Barcelona, Avenida Diagonal 643, 08014 Barcelona, Spain; 3 Otorhinolaryngology Department, Hospital Vall d’Hebron (HUVH), Passeig Vall d’Hebron 119-129, 08035 Barcelona, Spain; jlorente@vhebron.net; 4 Radiotherapy Unit, Vall d’Hebron Research Institute (VHIR), Autonomous University of Barcelona, Passeig Vall d’Hebron 119-129, 08035 Barcelona, Spain; sbenavente@vhebron.net; 5 Department of Otolaryngology-Head and Neck Surgery, Central University Hospital of Asturias, University of Oviedo, ISPA, IUOPA, 33011 Oviedo, Spain; juanagp@ispasturias.es (J.M.G.-P.); ynkc1@hotmail.com (E.A.); jprodrigo@uniovi.es (J.P.R.); 6 Spanish Biomedical Research Network Centre in Oncology (CIBERONC), Av. Roma SN, 33011 Oviedo, Spain; 7 Spanish Biomedical Research Network Centre in Oncology (CIBERONC), Vall d’Hebron Research Institute (VHIR), Passeig Vall d´Hebron 119–129, 08035 Barcelona, Spain

**Keywords:** cancer, HNSCC, resistance, cancer stem cells, apoptosis, autophagy

## Abstract

**Simple Summary:**

Therapy resistance in head and neck squamous cell carcinoma (HNSCC) patients is the main obstacle to achieve more effective treatments that improve survival and quality of life of these patients. Therefore, it is of vital importance to unravel the molecular and cellular mechanisms by which tumor cells acquire resistance to chemotherapy. We conducted a comparative proteomic study involving cisplatin-resistant cells and cancer stem cells with the aim of identifying proteins potentially implicated in the acquisition of cisplatin resistance. Through this study, we identified for the first time tetraspanin-1 (TSPAN1) as an important protein involved in the development, progression and chemoresistance of HNSCC tumors.

**Abstract:**

Sensitization of resistant cells and cancer stem cells (CSCs) represents a major challenge in cancer therapy. A proteomic study revealed tetraspanin-1 (TSPAN1) as a protein involved in acquisition of cisplatin (CDDP) resistance (Data are available via ProteomeXchange with identifier PXD020159). TSPAN1 was found to increase in CDDP-resistant cells, CSCs and biopsies from head and neck squamous cell carcinoma (HNSCC) patients. TSPAN1 depletion in parental and CDDP-resistant HNSCC cells reduced cell proliferation, induced apoptosis, decreased autophagy, sensitized to chemotherapeutic agents and inhibited several signaling cascades, with phospho-SRC inhibition being a major common target. Moreover, TSPAN1 depletion in vivo decreased the size and proliferation of parental and CDDP-resistant tumors and reduced metastatic spreading. Notably, CDDP-resistant tumors showed epithelial–mesenchymal transition (EMT) features that disappeared upon TSPAN1 inhibition, suggesting a link of TSPAN1 with EMT and metastasis. Immunohistochemical analysis of HNSCC specimens further revealed that TSPAN1 expression was correlated with phospho-SRC (pSRC), and inversely with E-cadherin, thus reinforcing TSPAN1 association with EMT. Overall, TSPAN1 emerges as a novel oncogenic protein and a promising target for HNSCC therapy.

## 1. Introduction

Head and neck squamous cell carcinoma (HNSCC) is a particularly aggressive cancer type since approximately 60% of patients have locally advanced disease upon diagnosis and approximately half of affected individuals do not survive more than five years after diagnosis [1]. Unfortunately, chemotherapeutic choices for the treatment of primary HNSCC have not improved over the past 50 years. Current chemotherapeutic options for HNSCC patients comprise chemotherapy, based mainly on cisplatin (CDDP) and also 5-fluorouracil, taxanes or cetuximab in certain metastatic tumors [2,3]. Recently, immunotherapeutic agents (nivolumab and pembrolizumab) have also been approved for recurrent/metastasic HNSCC [4]. In general terms, the high aggressiveness of HNSCC has been closely associated with the large genetic complexity and intratumor heterogeneity as well as the presence of distinct cancer cell subpopulations within the tumors showing different cancer stem cell (CSC) properties and resistance levels.

Resistance to stress, ultra-violet (UV) light, and chemotherapy or radiation treatment can be intrinsic or acquired. There is a general belief that resistance appears through natural selection of pre-existing mutant clones during stress. However, a novel type of resistance appears throughout the administration of chemotherapeutic agents, which seems to be related to treatment failure in cancer patients. Furthermore, the acquisition of resistance to one drug, usually favors the acquisition of resistance to other unrelated compounds, suggesting that the mechanisms of therapy resistance developed by cancer cells seem to follow a universal pattern against a wide variety of drugs [5,6].

CSCs are of particular relevance, emerging at the top level of the resistance scale since they are refractory to most, if not all, conventional chemotherapeutic treatments and radiotherapy. Accumulating studies show that various CSC markers and properties are crucial for tumor initiation, maintenance, expansion, and invasion, as well as mediators of treatment resistance, thereby allowing CSCs to survive in the most drastic conditions in vivo to further metastasize upon reaching an appropriate niche [7,8]. In particular, inhibition of key CSC markers such as Wnt, NANOG, CD44, CD133, CD55, ALDH1, OCT3/4, SOX2, KLF4 or NOTCH1 sensitizes cancer stem cells to chemotherapy [9,10]. Consistently, inhibition of CSC properties enhances the effects of chemotherapy [11].

Importantly, resistant phenotypes can be reverted to drug-sensitive phenotypes [12]. Consequently, in terms of applicability and clinical efficacy, cancer treatments should be capable of effectively targeting not only the bulk of cancer cells but also the subpopulations of drug-resistant cell clones, including CSCs.

In metabolomic and proteomic studies, our group has recently demonstrated common regulatory pathways to distinguish resistant cancer cells (non-CSCs) and CSCs from parental cells using a breast cancer model [13]. Hence, it is of major relevance to focus on those proteins present in resistant cancer cells and CSCs in order to design more effective treatment strategies that might synergistically act with standard chemotherapy [14]. With the idea of unraveling crucial proteins involved in HNSCC chemoresistance, a proteomic study was performed using the following cell lines: CCL-138 (parental), CCL-138-R (resistant to CDDP) and CSCs (derived from parental CCL-138 cells). Based on this analysis, we identified tetraspanin-1 (TSPAN1) as one of the most significantly upregulated proteins in CCL-138-R cells and CSCs compared to parental CCL-138 cells. Tetraspanins are a family of cell surface receptors that comprise a total of 33 members involved in signal transduction [15]. The role of tetraspanins in cancer has emerged in the last decade, being involved in proliferation, metastasis and immune protection and, consequently, postulated as potential oncogenic proteins [16,17]. Recently, some tetraspanin members have also been linked to chemoresistance. For example, TSPAN9 has been found to suppress chemosensitivity to 5-fluorouracil [18]. Specifically, TSPAN1 has been proved to be important for the development of prostate, colon and gastric cancer [17,19]. For the first time, this study investigates the clinical and biological relevance of TSPAN1 in HNSCC, with a special focus on laryngeal and pharyngeal tumors. For this purpose, the impact of TSPAN1 inhibition was functionally assessed both in vitro and in vivo using disease-relevant HNSCC models, and the clinical role of TSPAN1 was also further explored in a large series of HNSCC patient samples.

## 2. Materials and Methods

### 2.1. Cell Lines and Culture Conditions

Fresh samples of normal and tumor tissue from patients surgically treated for squamous larynx carcinoma were obtained from the Otorhinolaryngology department of Vall d´Hebron University Hospital (HUVH) as described [20]. These patients had not previously undergone any radio- or chemotherapeutic treatment before surgical intervention. All of them signed the informed consent for the analysis and publication of the data derived from their samples. The present study was carried out following the instructions and requirements of the declaration of Helsinki along with the approval of the Ethical Committee of the HUVH (Ref: PR[AG]342/2016). Patient-derived cell lines were established in culture.

The human pharyngeal cancer cell lines HTB-43 (FaDu) and CCL-138 (Detroit 562) were purchased from the American Type Culture Collection (ATCC, Manassas, VA, USA), while the laryngeal cancer cell line JHU029 Lucires-GFP (RRID: CVCL_5993) and the tongue cell line SSC-25 were kindly provided by Dr. S. Aznar-Benitah (Institute for Research in Biomedicine Barcelona, Spain). All of them were satisfactorily authenticated based on polymorphism of small repetitive tandem loci. The above-mentioned HNSCC cell lines and their respective Cancer Stem Cells (CSCs) subpopulations were cultured as previously described [20].

### 2.2. Drug Assay

To determine their respective IC50, JHU029, JHU029-R, HTB-43, HTB-43-R, CCL-138 and CCL-138-R, cell lines were seeded at 15 × 10^3^ cells per well into 96-well plates, and the primary cell lines derived from patients at 10 × 10^3^ cells per well. Twenty-four hours later, cells were exposed in triplicate to CDDP (range 0–150 μM) or dasatinib (Sigma-Aldrich, Merck, Madrid, Spain) (0–3 μM) for 48 h. Cellular viability was measured by MTS (AQueous MTS Reagent Powder, Promega Biotech Iberica, Madrid, Spain) based on the rate of metabolic activity of the previously treated cells.

### 2.3. Proteomic Study

A comparative proteomic analysis of the following cell lines was performed: CCL-138, CCL-138-R and CCL-138 CSCs by using three biological replicates for each of them. The proteomic study has been carried out by the COS Centre (Centre for Omic Sciences, Tarragona, Spain).

### 2.4. Sample Preparation and LC-MS/MS Analyses

The proteomic study was carried out by the technological infrastructures of the Center for Omic Sciences (COS, EURECAT, Tarragona, Spain). Cell lysis and protein solubilization was performed with modified Pierce RIPA Buffer (Thermo Scientific, Waltham, MA, USA). Thus, 200 µL of lysis buffer (25 mM Tris-HCl (pH 7.6), 150 mM NaCl, 1% NP-40, 1% sodium deoxycholate, 1% SDS and protease and phosphatases inhibitors) was added to cell pellets that were sonicated (Sonics & Materials, Vibra Cell, Illkirch, France) for three cycles of 10 s repeated six times with 15-s pause intervals. Afterwards, the suspension was centrifuged, the supernatants transferred to new tubes and protein concentration was determined by the Lowry method. Then, 65 μg of protein from each sample was reduced with 4 mM DTT for 25 min at 56 °C and alkylated with 8 mM iodoacetamide for 30 min at 25 °C in the dark and loaded in a polyacrylamide gel to remove detergents. Next, the gel slice containing unresolved proteins was cut into small pieces and digested overnight at 37 °C with trypsin at an enzyme: protein ratio of 1:100. After digestion, a small aliquot of 2 μg was purified using C18 zip-tip (Merck Life Science, Madrid, Spain) to check proper protein digestion by nanoLC-Orbitrap before following sample preparation steps.

### 2.5. Peptide 10-Plex TMT Labeling

The remaining digested protein sample was desalted on C18 Sep-Pack column (Waters, Bedford, MA, USA) using 80% acetonitrile, 20% water with 0.1% formic acid for elution. The eluted peptides were dried in the Speed-Vac and labeled with TMT 10-plex labelling (Thermo Fisher Scientific, Waltham, MA, USA) following manufacturer’s instructions.

To normalize all the samples, a pool was created by mixing an equally small aliquot of each sample and then, 40 μg of this pool and 40 μg of each of the nine individual samples were labeled. Next, a small aliquot of 5 μg from each labeled sample was mixed and purified using C18 zip-tip (Millipore, Burlington, MA, USA) and analyzed by nanoLC-Orbitrap to check the labeling reaction.

### 2.6. Peptide Fractionation

The labeled peptides from each sample were mixed together and desalted on C18 Sep-Pack column (Waters, Bedford, MA, USA) using 80% acetonitrile, 20% water with 0.1% formic acid for elution. The TMT pooled sample was dried in the Speed-Vac and resuspended in rehydration buffer (5% Glycerol and 1% *v/v* IPG strip Buffer 3-10NL), and subsequently fractionated by isoelectrofocusing on an Off-Gel fractionator from Agilent Technologies through 12-well IPG strips (Nonlinear gradient from pH 3 to 10) according to the supplier’s protocol. Initially, 13-cm-long IPG strips were hydrated with 40 μL per well of the rehydration buffer. 200 μg of TMT pooled sample was loaded on the strip (150 μL of sample in each well). The samples were focused at 50 μA, with voltages between 500 and 4500 V for a total of 20 kVh. After separation, each one of the 12 fractions obtained was desalted on C18 Sep-Pack column (Waters, Bedford, MA, USA) using 80% acetonitrile, 20% water with 0.1% formic acid for elution. Eluted fractions were resuspended in 50 μL of 0.1% formic acid.

### 2.7. NanoLC-(Orbitrap) MS Analysis

The 12 fractions obtained from Off-Gel fractionation method were separated on a trap nano-column (100 μm I.D.; 2 cm length; 5 μm particle diameter, Thermo Fisher Scientific, San José, CA, USA), and then separated onto a C-18 reversed phase (RP) nano-column (75 μm I.D.; 15 cm length; 3 μm particle diameter, Nikkyo Technos Co. LTD, Tokyo, Japan). The chromatographic separation was performed with a continuous acetonitrile gradient using Milli-Q water (0.1% FA) and ACN (0.1% FA) as mobile phase. A flow rate of 300 nL/min was used to elute peptides for real time ionization and peptide fragmentation on an LTQ-Orbitrap Velos Pro mass spectrometer (Thermo Fisher). An enhanced FT-resolution spectrum (resolution = 30,000 FHMW) followed by two data dependent MS/MS scan events was performed. One consists of an HCD fragmentation (40% NCE) and FT-MS/MS acquisition (R = 15,000 FHMW) from most intense ten parent ions with a charge state rejection of 1 and dynamic exclusion of 0.5 min, which is used for peptide quantification. The other event consists of a CID fragmentation (35% NCE) and IT-MS/MS acquisition from the same most intense ten parent ions, which is used for peptide identification. The 12 raw data files obtained were analyzed by Multidimensional Protein Identification Technology (MudPIT) on Proteome Discoverer software v.1.4.0.288 (Thermo Fisher Scientific). For protein identification, all MS and MS/MS spectra were analyzed using Mascot search engine (version 2.5). Mascot was set up to search the SwissProt_2017_05.fasta database (554,515 entries), restricting for human taxonomy (20,202 sequences) and assuming trypsin digestion. Two missed cleavages were allowed and an error of 0.02 Da for FT-MS/MS fragment ion mass, 0.8 Da for IT-MS/MS fragment ion mass and 10.0 ppm for a FT-MS parent ion mass were allowed. TMT-10plex on lysine and N-termini were set as quantification modifications, oxidation of methionine and acetylation of N-termini were set as dynamic modifications, whereas carbamidomethylation of cysteine was set as static modifications. The false discovery rate (FDR) and protein probabilities were calculated by Percolator software.

The mass spectrometry proteomics data have been deposited to the ProteomeXchange Consortium via the PRIDE partner repository [21], dataset identifier PXD020159.

### 2.8. Protein Analysis

Total protein extracts used for Western blot analysis were obtained from subconfluent cells and lysed with RIPA buffer (25 mM TrisCl, 150 mM NaCl, 1% Igepal, 1% sodium deoxycholate, 0.1% SDS, pH 7.5, plus 2 mM full cocktail of protease and phosphatases inhibitors, Thermo Scientific). The following primary antibodies were incubated overnight at 4 °C: TSPAN1 (ab96070), ATG5 (ab109490) (Abcam, Cambridge, UK); LC3B (#3868), pScr (#6943), Src (#2109), pAKT (# 9271), AKT (#9272), PARP1 (#9542S), pERK1/2 (#9101L) (Cell Signaling Technology Europe Leiden, The Netherlands); SQSTM1 (p62) (SAB3500430), β-actin (A3854) (Sigma—Aldrich Química SL, Madrid, Spain); ERK1/2 (sc-514302), Vinculin (sc-73614) (Santa Cruz Biotechnology, Heidelberg, Germany). The membranes were revealed with Super Signal™ West Pico PLUS Chemiluminescent Substrate (Thermo Scientific). Results were confirmed in at least three independent experiments.

### 2.9. Transfections

Two hundred thousand HNSCC cells were transfected in reverse with 10 nM siRNA TSPAN1 (siTSPAN1) or 10 nM siRNA negative control (NC) (Integrated DNA Technologies) and 4 µL of Lipofectamine RNAimax (Invitrogen, Thermo Fisher Scientific, Waltham, MA, USA) per well for 6-well plates in OPTIMEM serum-free medium (Gibco, Thermo Fisher, Waltham, MA, USA). Extracts were collected at 48–72 h after transfection. Furthermore, we used an additional siTSPAN1 (siTOOLs Biotech, Planegg, Germany) to demonstrate independently the biological effect of TSPAN1 inhibition. This siRNA complex pool (siPOOLs) consists of 30 optimally-designed siRNAs that has demonstrated to efficiently remove off-target effects and improve reliability of the results [22]. For the construction of the corresponding proliferation curves, six cell counts were performed every 1–3 days up to a maximum of 12 days.

### 2.10. Colony Formation Assay

A total of 5 × 10^3^ cells were seeded per well in triplicate in 6-well plates and the colony formation was allowed to grow for 10–14 days. The quantification of the colonies was performed by fixation with 4% paraformaldehyde, followed by staining with crystal violet (0.5% *w/v* in water) and quantified colorimetrically (once treated with 15% acetic acid), at 595 nm in an Epoch spectrophotometer (Biotek, Winooski, VT, USA). Results were confirmed in at least three independent experiments.

### 2.11. Quantitative Real-Time PCR (qRT-PCR)

Total RNA was extracted from transfected cells at 72 h post-transfection using MirVana kit (Ambion, Austin, TX, USA). Subsequently, total RNA was treated with DNAse I using the DNA-free™ DNA Removal kit (Invitrogen, Thermo Fisher Scientific), according to the manufacturer’s instructions. The cDNA was obtained from PCR amplification of 500 ng of total RNA (RIN ratio ≥ 8) using the RevertAid H Minus First Strand cDNA Synthesis kit. The expression of TSPAN1 (Hs00371661_m1) was analyzed by qRT-PCR using the expression of IPO8 (Hs00183533_m1) as an endogenous gene and TaqMan™ Universal Master Mix II (Applied Biosystems, CA, USA; Thermo Fisher Scientific). Results were confirmed in at least three independent experiments (each sample run in triplicate).

Apoptosis detection by flow cytometry and autophagy analysis by transmission electron microscopy (TEM), were respectively described elsewhere [20,23].

### 2.12. Animal Models

Five-week-old female mice from NMRI-FOXn1 nu/nu strain (Janvier Labs, Saint Berthevin Cedex, Le Genest-Saint-Isle, France) were used for in vivo studies. JHU029 and JHU029-R cell lines were both labeled with luciferase and GFP as previously described [24], and transfected with siTSPAN1 vs. negative control (NC). Forty-eight hours post-transfection, 1 × 10^6^ cells were injected in each flank of each animal, at a final volume of 200 µL of PBS and Matrigel (1:1, *v:v*) (Life Sciences, Corning, NY, USA). A total of 16 mice were included in the study, four mice for each of the following experimental groups: JHU029 NC, JHU029 siTSPAN1, JHU029-R NC, JHU029-R siTSPAN1. Tumor volume was measured every 2–4 days, for a total of 42 days, using an electronic calibrator. At the end point, the animals were analyzed by IVIS Spectrum (Perkin Elmer, Waltham, MA, USA). Mice were sacrificed by cervical dislocation. An autopsy was performed on all the animals to collect liver, spleen, kidneys, lungs, heart and bones, which were analyzed by IVIS Spectrum for ex vivo detection of micrometastases.

### 2.13. Image Platform

Optical imaging studies were carried out by the Preclinical Imaging Platform technicians (Lab Animal Service, Campus Vall d’Hebron, Barcelona, Spain). For bioluminescence studies, animals were injected with an intraperitoneal injection of luciferin at a dose of 150 mg/kg body weight. Then, the animals were anesthetized prior to the scans with an isoflurane mixture (5% during induction, 2% in maintenance). Air flow was 0.8 L/min. After the image acquisitions, the animals were returned to their cages for recovery. All the procedures were performed following instructions from the institutional ethic committee (Ref. CEEA 42/15). Images were analyzed by the Preclinical Imaging Platform staff with Living Image^®^ software, from Perkin Elmer. Study analysis consists in light radiance quantification. Signals from the light sources were isolated and characterized. The kinetic curve of bioluminescence chemical reaction was examined, searching for the highest signals in each light source. These signals are theoretically proportional to the number of cells involved in the bioluminescence reaction. Analysis units were photons (p)/second (s)/centimeter (cm^2^) /stereoradian (sr). These units are technically corrected and allow comparisons between different studies.

### 2.14. Patients

Surgical tissue specimens, from 106 patients with laryngeal or pharyngeal squamous cell carcinoma who underwent surgical treatment at the Hospital Universitario Central de Asturias, were retrospectively collected in order to perform immunohistochemistry (IHC) studies. All experimental procedures were conducted in accordance to the Declaration of Helsinki and approved by the Institutional Ethics Committee of the Hospital Universitario Central de Asturias (Ref. 141/19, project PI19/00560). Representative tissue sections were obtained from archival, paraffin-embedded blocks, and the histological diagnosis was confirmed by an experienced pathologist. Three morphologically representative areas were selected from each individual tumor block to construct five tissue microarray (TMA) blocks. In addition, each TMA also contained three cores of normal epithelium as an internal control. All patients had a single primary tumor, microscopically clear surgical margins and received no treatment prior to surgery. All patients were habitual tobacco smokers, 65 moderate (1–50 pack/year) and 41 heavy (>50 pack/year); 98 were habitual alcohol drinkers and 57 out of 106 patients (54%) received postoperative radiotherapy. The clinicopathologic features of HNSCC patients are summarized in Table S1. The stage of disease was determined after the surgical resection of the tumor according to the TNM system of the International Union against Cancer (7th Edition). The histological grade was determined according to the degree of differentiation of the tumor (Broders’ classification).

### 2.15. IHC

The formalin-fixed, paraffin-embedded tissues were cut into 3-μm sections and dried on Flex IHC microscope slides (Dako, Glostrup, Denmark). The sections were deparaffinized with standard xylene and hydrated through graded alcohols into water. Antigen retrieval was performed using Envision Flex Target Retrieval solution (Dako, Glostrup, Denmark), high pH. Staining was done at room temperature on an automatic staining workstation (Dako Autostainer Plus), using the Dako EnVision Flex + Visualization System (Dako Autostainer, Denmark) with the following antibodies: anti-TSPAN1 monoclonal antibody Clone HPA011909 (Sigma—Aldrich Química SL, Madrid, Spain) at 1:50 dilution, anti-E-Cadherin (BD Biosciences #610181, San Jose, CA, USA) at 1:4000 dilution and active SRC monoclonal antibody Clone 28 (Thermo Fisher Scientific #AHO0051) at 1:300 dilution. The SRC antibody, which detects the active protein, has been previously described [25]. Counterstaining with hematoxylin was the final step. Immunostaining was scored blinded to clinical data by two independent observers. TSPAN1 and E-cadherin were scored as follows: quantity scores from 0 to 3 were respectively assigned if 0%, 1% to 10%, 11% to 50%, and 51% to 100% of the tumor cells showed cytoplasmic staining, and the staining intensity was rated on a scale of 0 to 2 (0 = negative, 1 = weak, 2 = strong). The raw data were then converted to an Immunoreactive Score (IRS) by multiplying the quantity and staining intensity scores. For E-cadherin, an IRS score equal to or above the median (score 4) was considered high expression. TSPAN1 expression was dichotomized as negative (score 0) vs positive (scores 1 to 6) expression. In addition, nuclear expression of TSPAN1 was recorded as negative vs. positive. Active SRC staining showed a homogeneous distribution, and therefore a semiquantitative scoring system based on staining intensity was applied: low (0, 1+), moderate (2+), or high expression (3+).

### 2.16. Experimental Design and Statistical Rationale

In order to discover new proteins involved in HNSCC chemoresistance, a comparative proteomic analysis was performed using CDDP-resistant cells (CCL-138-R) and CSCs in relation to control cells (CCL-138). For protein quantification, the ratios between each TMT-label against 126-TMT label were used. Protein quantification was normalized based on protein median and Log2 transformed for statistical analysis. Statistical analysis was performed by using Mass Profiler Professional software v. 14.5 from Agilent Technologies to find significant protein changes between the cell conditions assayed. Two different statistical analyses were performed to cover the different experimental conditions described. In first instance, One-way ANOVA and Post-Hoc analysis was applied to all cell groups. Next, an unpaired T-test for CCL-138 CSCs vs CCL-138 and CCL-138-R vs CCL-138 was applied to check differences between them. In both cases, a Benjamini-Hochberg *p*-value correction for multiple comparisons was applied to reduce false-positive findings. Only those proteins that were quantified in at least 2 out of 3 independent experiments were considered for statistical purposes.

To determine the relevance of TSPAN1 expression in both HNSCC cell lines and patients including its possible relevance in the clinic, all the statistical analyses in patient samples and cell lines were performed using the SPSS 15.0 software package (SPSS Inc., Chicago, IL, USA). The χ² test and Fisher’s exact test were used for comparison between categorical variables. For time-to-event analysis, Kaplan–Meier curves were plotted. Differences between survival times were analyzed by the log-rank method. Wilcoxon test was used as a non-parametric test for mean ranks comparisons. Unpaired Student’s *t*-test was performed to compare means between two groups. All tests were two-tailed. *p* values below 0.05 were considered statistically significant.

## 3. Results

### 3.1. TSPAN1 Protein Is Upregulated in CDDP-Resistant HNSCC Cells

**Figure 1:**
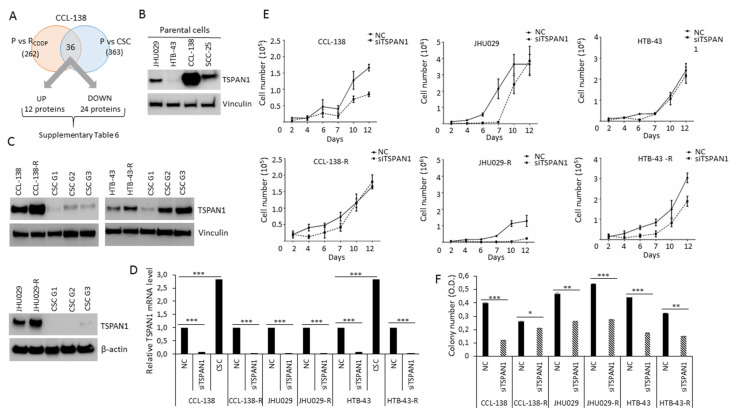
TSPAN1 is revealed as a protein involved in head and neck squamous cell carcinoma (HNSCC) chemoresistance and proliferation. (**A**) The proteomic study identified 36 proteins commonly deregulated in CSCs and CDDP-resistant cells compared with parentals (P) cells, TSPAN1 was one of the most highly upregulated protein. (**B**) Western blot analysis of TSPAN1 expresion in four different HNSCC cells (CCL-138, JHU029, HTB-43 and SCC-25). (**C**) Western blot analysis confirmed proteomic data verifying increased TSPAN1 expression in CDDP-resistant cells; and CSC G2 and G3 from the HTB-43 cell line. (**D**) TSPAN1 mRNA levels in parental and CDDP-resistant HNSCC cells upon TSPAN1 depletion. Data were normalized to IPO8 (endogenous control), and relative to NC (negative control or scramble) cells assigned the value of 1. TSPAN1 mRNA level for CSC from HTB-43 and CCL-138 cell lines was also assessed. Unpaired two-tailed Student’s *t*-test, *** (*p* < 0.001). (**E**) Proliferation curves of cells transduced with siTSPAN1 vs. non-targeted siRNA control (NC) are shown. (**F**) Colony number quantification of HNSCC cell lines transduced with siTSPAN1 compared to the respective NC controls. Unpaired two-tailed Student’s *t*-test, * (*p* < 0.05), ** (*p* < 0.01), *** (*p* < 0.001). OD: Optical Density measured at 595 nm (Crystal violet).

In order to identify proteins that might potentially have a relevant role in cancer chemoresistance, a proteomic analysis was performed to compare the proteins expressed in parental CCL-138 cells vs. CSCs and CCL-138-R cells (CDDP-resistant cells). A total of 1535 proteins were identified in these three cell groups (Table S2). One-way ANOVA showed that 476 proteins were differentially expressed among at least two of these groups (Table S3). A Post-Hoc analysis revealed which of these proteins were dysregulated in each group, by comparing CCL-138-R vs. CCL-138 (262 proteins), and CSC vs. CCL-138 (363 proteins) (Tables S4 and S5). Finally, 36 proteins were commonly and differentially expressed in CCL-138-R and CSCs compared to parental CCL-138 cells; 12 proteins were upregulated and 24 proteins downregulated (Figure 1A, Table S6). One of the most upregulated protein, a member of the tetraspanin family, named TSPAN1, was primarily selected for its potential clinical interest in various cancer models [26,27]. In order to explore the putative relevance of TSPAN1 in HNSCC, TSPAN1 levels were compared in the following HNSCC cell lines: CCL-138, JHU029, HTB-43 and SSC-25. The metastatic cell line CCL-138 derived from a pharyngeal cancer showed the highest TSPAN1 levels (Figure 1B, Figure S8). In order to verify the proteomic results, TSPAN1 expression was analyzed by Western blot in the aforementioned HNSCC cell lines confirming that TSPAN1 was upregulated in CCL-138-R, JHU029-R and HTB-43-R cells compared to their respective control cells (Figure 1C, Figure S8). Moreover, TSPAN1 was upregulated in the second and third generation (G2 and G3) of enriched CSCs in HTB-43 cells at protein level and also in G3 from HTB-43 and CCL-138 at mRNA level, as compared to parental cells (Figure 1C,D). It should be noted that HTB-43 cell line has the highest proliferative capacity in 3D conditions [28].

The functional relevance of TSPAN1 was assessed by siRNA depletion in HNSCC cells. Transduced parental JHU029, HTB-43 and CCL-138 cells and their respective CDDP-resistant variants with siTSPAN1 robustly decreased TSPAN1 mRNA levels by ~90% in all cell lines (Figure 1D). Cell proliferation decreased in TSPAN1-depleted cells (Figure 1E), and the most potent reduction was observed in resistant JHU029-R and HTB-43-R cells compared to parental cells (Figure 1E and Figure S1). Consistently, colony formation capacity was also significantly reduced upon TSPAN1 depletion in CDDP-resistant and parental HNSCC cells (Figure 1F).

### 3.2. TSPAN1 Inhibition Induces Sensitivity of HNSCC Cells to Chemotherapeutic Agents

**Figure 2:**
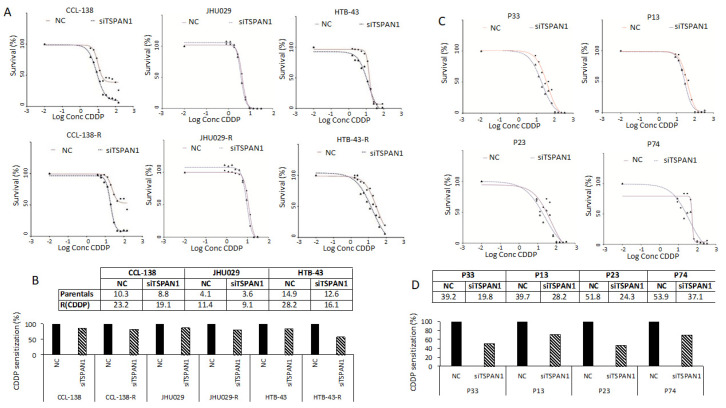
TSPAN1 sensitizes HNSCC cells to CDDP. (**A**) TSPAN1 depletion sensitizes parental and resistant cells CCL-138, JHU029 and HTB-43 to CDDP. A representative experiment is shown from at least three independent experiments. (**B**) Table summarizes the IC50 values (µM) from the plots shown in panel A, the IC50 values are represented graphically as the percentage of sensitization relative to control cells (assigned a value of 100%). (**C**) Sensitization of biopsy-derived HNSCC cell lines from four CDDP-resistant cancer patients: P33, P13 and P74 (laryngeal biopsies) and P23 (pharyngeal biopsy). A representative experiment is shown from at least three independent experiments. (**D**) Table summarizes the IC50 values (µM) from the plots shown in panel C, the IC50 values are represented graphically as the percentage of sensitization relative to control cells (assigned a value of 100%).

The effects of TSPAN1 depletion on drug response were analyzed in parental and resistant JHU029, HTB-43 and CCL-138 cells. These cell lines showed levels of sensitization to CDDP that vary between 14–43% IC50 reductions in relation to the control cells. Sensitization to CDDP was observed by TSPAN1 depletion in all cell lines, with a higher effect in the respective resistant variants, and HTB-43-R cells showing the highest sensitization (Figure 2A,B). In order to demonstrate that the phenotypic effects observed by TSPAN1 inhibition using the previous siRNA was not due to off-target effects, we tested an independent siRNA#2 against TSPAN1 (Integrated DNA Technologies, Coralville, IA, USA). Moreover, a pool of 30 specific siRNAs against TSPAN1 (siPool) (siTOOLsBiotech, Planegg, Germany) was also tested. TSPAN1 inhibition with these new siRNAs (siRNA#2 and siPool) decreased cell proliferation and sensitized HNSCC cells to the effects of CDDP, corroborating our previous results (Figure S2) [28]. The role of TSPAN1 on CDDP response was also explored in biopsy-derived cell lines from laryngeal and pharyngeal cancer patients. We chose four biopsy-derived cell lines with high levels of resistance to CDDP (IC50 values ranging 39–53 µM). According to our previous report, IC50 values >15 µM were considered as highly resistant to CDDP [20]. All four biopsy-derived cell lines showed CDDP sensitization upon TSPAN1 inhibition, with a range of 29–53% IC50 reductions in comparison with control cells (Figure 2C,D). Therefore, TSPAN1 depletion sensitizes with a stronger effect in biopsy-derived cell lines than in established CDDP-resistant HNSCC cell lines.

Next, we investigated if the sensitizing action of TSPAN1 depletion could be extended to other therapeutic drugs, such as dasatinib, a dual SRC/ABL kinase inhibitor actively tested in various clinical trials, including HNSCC [29,30] (www.clinicaltrials.gov). TSPAN1 inhibition sensitized HNSCC cells to dasatinib (Figure S3). Hence, the effect of TSPAN1 inhibition on drug sensitization is not restricted to CDDP and, beyond dasatinib, it could also extend to other chemotherapeutic drugs.

### 3.3. TSPAN1 Depletion Induces Apoptosis in HNSCC Cells

**Figure 3:**
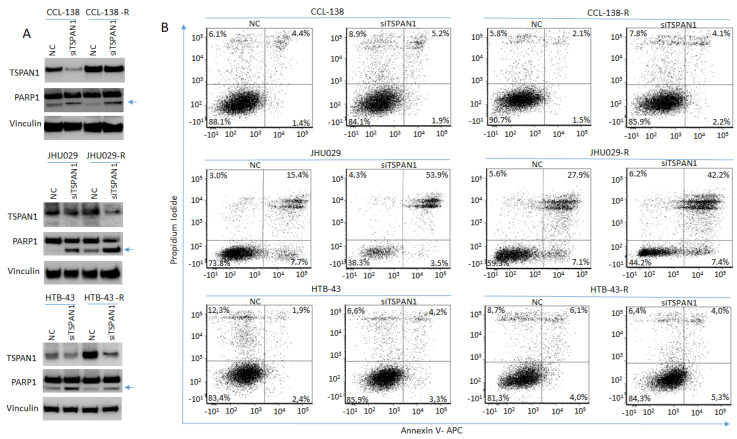
TSPAN1 inhibition induces apoptosis in HNSCC cells. (**A**) Western blot analysis of TSPAN1 and PARP1 cleavage in parental and CDDP-resistant HNSCC cells upon TSPAN1 depletion. In the CCL-138-R cell line, despite not observing TSPAN1 inhibition at protein level (but it was inhibited at the mRNA level), an induction of PARP1 was observed. Note: the lower band corresponds to cleaved PARP1 (arrow). Vinculin was used for protein loading normalization. (**B**) Apoptosis analysis by FACS using Annexin V expression kit. Representative scatter plots of propidium iodide (*y*-axis) vs. Annexin V-APC (*x*-axis) to detect early (right bottom quadrant) and late (right upper quadrant) apoptosis in parental and their respective CDDP-resistant cells (-R) JHU029, HTB-43 and CCL-138, comparing NC vs. TSPAN1depleted cells.

**Figure 4:**
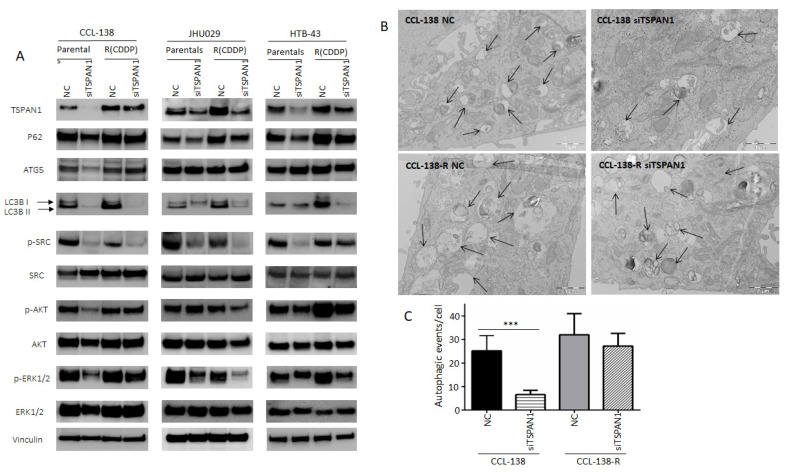
Characterization of TSPAN1 downstream effectors. (**A**) Western blot analysis of proteins modulated by TSPAN1 depletion in HNSCC cells, including various key proteins related to autophagy and SRC/AKT/ERK signaling pathways. (**B**) Representative electronic microscopy images in TSPAN1-depleted cells vs. control cells (NC) in CCL-138 and CCL-138-R cells. TEM images revealed autophagosomes and autophagolysosomes, characteristic features of autophagic cells. (**C**) Quantification of autophagic vesicles from each experimental group. Note that autophagy vesicles decrease in CCL-138 cells upon TSPAN1 depletion. Unpaired two-tailed Student’s *t*-test, *** (*p* < 0.001).

TSPAN1 protein levels were effectively reduced by siTSPAN1 (Figure 3A, Figure 4A and Figure S8). Evidence of apoptosis induction upon TSPAN1 inhibition emerged from PARP cleavage analysis by Western blot (Figure 3A and Figure S8). The active form of PARP, cleaved PARP1 -indicative of apoptosis activation-, was found to increase in all TSPAN1-depleted cell lines. To corroborate these results, apoptosis was further evaluated by Annexin V through flow cytometry (FACS). Upon TSPAN1 inhibition, apoptosis increased in all cell lines except HTB-43-R, which was only observed in early apoptosis, (Figure 3B). In CCL-138 cells, the early and late apoptosis represent 1.4% and 4.4%, respectively (5.8% total), whereas these percentages slightly increased to 1.9% and 5.2% (7.1% total) in the corresponding TSPAN1-depleted cells. For CCL-138-R cells, early and late apoptosis represent 1.5% and 2.1%, respectively (3.6% total), while apoptosis further increased in TSPAN1-depleted cells to reach 2.2% and 4.1%, respectively (6.3% total). In parental JHU029 cells, early and late apoptosis were 7.7% and 15.4%, respectively (23.1% total apoptosis), robustly increasing in TSPAN1-depleted cells up to 3.5% and 53.9% (57.4% total). Similarly, early and late apoptosis in resistant JHU029-R cells represent 7.1% and 27.9%, respectively (35% total), and apoptosis was markedly induced in TSPAN1-depleted JHU029-R cells, reaching 7.4% and 42.2% (49.6% total). For parental HTB-43 cells, the early and late apoptosis represent 2.4% and 1.9% respectively (4.3% total), respectively increasing to 3.3% and 4.2% (7.5% total) by TSPAN1 depletion. For HTB-43-R cells, early and late apoptosis represent 4.0% and 6.1% respectively (10.1%) compared to 5.3% and 4.0% (9.3%) in TSPAN1-depleted cells (Figure 3B). These results show that TSPAN1 depletion induces apoptosis in HNSCC cells.

### 3.4. TSPAN1 Depletion Downregulates Several Signaling Cascades with SRC Kinase Signaling as a Central Node

Based on our previous report linking HNSCC chemoresistance and autophagy [20], the possible relationship between TSPAN1 and autophagy was investigated. LC3 lipidated (LC3-II) (the most universal marker of autophagy activation) and p62 proteins decreased at protein level in cells depleted for TSPAN1 (ATG5 only decreased in parental CCL-138 cells) (Figure 4A and Figure S8). Autophagy inhibition was confirmed by TEM (Figure 4B,C). Therefore, this inhibitory effect on autophagy might be associated with the suppressive effect of TSPAN1 depletion on cell proliferation and apoptosis induction. Given the fact that TSPAN1 inhibition sensitized both parental and the CDDP-resistant derivatives of JHU029, HTB-43 and CCL-138 cells to dasatinib (Figure S3), we wonder whether phosphorylation of SRC kinase (p-SRC or active SRC), a direct target of dasatinib, could be a mediator of TSPAN1 function in our HNSCC models. In fact, p-SRC levels (an indicator of SRC activation) consistently decreased in all six cell lines tested: JHU029, JHU029-R, HTB-43, HTB-43-R, CCL-138, CCL-138-R upon TSPAN1 depletion (Figure 4A). Notably, p-SRC levels correlate well with TSPAN1 expression and, although expression is low in CSCs at G1, both proteins increase in CSCs at G3 (Figure S4A,B). Interestingly, dasatinib robustly inhibited SRC activity and its major target p-SRC in our HNSCC models; however, dasatinib did not alter the expression of various autophagy-related proteins or TSPAN1 (Figure S4B). These findings indicate that TSPAN1 inhibition efficiently targets SRC-dependent and independent signaling pathways, beyond merely mimicking the action of dasatinib as a SRC inhibitor.

It has also been recently described that TSPAN1 promotes epithelial-to-mesenchymal transition (EMT) and metastasis in a cholangiocarcinoma cancer model [31]. This prompted us to study the link between TSPAN1 and EMT in our HNSCC models. For this purpose, various EMT-related proteins vimentin, E-cadherin and N-cadherin were analyzed by Western blot in TSPAN1-depleted cells vs. NC. Only the expression of vimentin (a mesenchymal-related protein) decreased upon TSPAN1 inhibition in JHU029 and JHU029-R cells, but no clear differences were observed in E-cadherin or N-cadherin, nor in the other HNSCC cell lines [28]. Wang et al. [31] described that TSPAN1 promotes EMT acting through ERK1/2 and AKT pathways. Western blot analysis of p-ERK1/2 and p-AKT was hence performed to explore this possibility in our HNSCC models. p-AKT levels were found to decrease in CCL-138, JHU029-R, HTB-43 and HTB-43-R cells upon TSPAN1 inhibition. p-ERK1/2 also decreased at varying levelsin CCL-138, CCL-138-R, JHU029, JHU029-R, HTB-43 and HTB-43-R cells (Figure 4A). In JHU029-R, CCL-138 and HTB-43 cells, dasatinib treatment decreased p-ERK1/2, but did not affect p-AKT levels (Figure S4B).

### 3.5. TSPAN1 Depletion Decreases Tumor Formation and Metastatic Capacity in Mice

**Figure 5:**
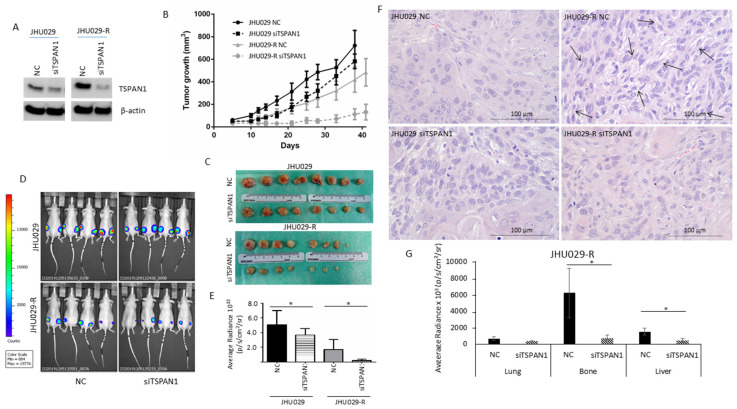
Impact of TSPAN1 inhibition in vivo. (**A**) JHU029 and JHU029-R cells were transduced with siTSPAN1 and injected into immunosuppressed mice. TSPAN1 inhibition was monitored in cultured cells at day 2 after transfection. (**B**) Graph representing the growth of mice tumors originated from the indicated cell lines upon TSPAN1 inhibition vs. negative controls (NC). Data are expressed as mean ± SD. Significant differences were observed between JHU029 NC vs siTSPAN1 (days 10–28), and JHU029-R NC vs siTSPAN1 (days 12–41). Unpaired Student’s *t*-test. (**C**) Pictures of the tumors formed in the indicated group of mice. (**D**) Pictures of the mice tumors at the end point of the experiment. The luminescent signal was taken by the IVIS apparatus. (**E**) Quantification of the luminescent signals of tumors were acquired by Living Image software, and graphed. Unpaired two-tailed Student’s *t*-test, * (*p* < 0.05). (**F**) Representative images of H&E staining for the indicated mice tumors. Note the presence of a fusocellular pattern (arows) in the resistant cells but not in parental cells. The fusocellular pattern, indicative of EMT activation, was reverted in the TSPAN1-depleted tumor subgroup (JHU029-R-NC vs. JHU029-R-siTSPAN1). Scale bar: 100 µm (**G**) Quantification of micrometastases ex vivo based on luminescent signals acquired by Living Image software in the indicated organs from the JHU029-R mice tumors. Unpaired two-tailed Student’s *t*-test, * (*p* < 0.05).

The role of TSPAN1 was investigated in HNSCC models in vivo, inducing mice tumors with TSPAN1-depleted JHU029 and JHU029-R cells vs. control cells (NC). In such cells, TSPAN1 depletion was confirmed (pre-injection) at protein level (Figure 5A and Figure S8). Tumors formed by JHU029 cells phenotypically reproduced the characteristics observed in HNSCC patients (Figure S5A). Tumor sizes were significantly lower in the tumors generated from TSPAN1-depleted cells compared to those formed by control cells (Figure 5B,C and Figure S5B). Measurement of tumor size by IVIS bioluminescence imaging further confirmed these results (Figure 5D,E). Notably, tumors formed by the resistant JHU029-R cells, despite being smaller in size, showed EMT features, characteristic of fusocellular morphology (Figure 5F, arrows). Moreover, the EMT phenotypes were reversed in tumors formed by TSPAN1-depleted JHU029-R cells. At the endpoint of the experiment, we found that tumors from the group JHU029-R siTSPAN1 generally showed lower TSPAN1 expression than the corresponding control group (JHU029-R NC) (Figure S5C), beyond some variability in TSPAN1 levels among the tumors from each experimental group. The pathological examination of the primary tumors generated in mice showed no significant differences in Ki-67 staining regardless TSPAN1-depletion (Figure S5D). The impact of TSPAN1 inhibition on metastasis formation was also evaluated ex vivo, thereby monitoring the presence of luciferin-positive JHU029-R cells in various organs extracted from the different mice using IVIS bioluminescence imaging system (Figure S6). A significant decrease in bone and liver metastasis was measured in tumors originated from TSPAN1-depleted JHU029-R cells compared to control cells, and a trend to significance in lung metastasis was also observed (Figure 5G). Overall, TSPAN1 inhibition renders smaller tumors with reduced metastatic potential.

### 3.6. TSPAN1 Expression in Patient Samples. Correlations with Active SRC and EMT Features

The relevance of TSPAN1 expression was further investigated in patient biopsies. Firstly, the expression of TSPAN1 was analyzed at mRNA level in 16 biopsies of tumor tissue and patient-matched normal mucosa. In eight out of 16 patients, TSPAN1 mRNA was upregulated (50% of cases) (Figure S7A,B). In silico analysis of TCGA database was performed to explore TSPAN1 mRNA expression more extensively in different cancer types [32]. It was revealed that TSPAN1 mRNA was frequently and commonly upregulated in multiple cancers compared to the corresponding normal counterparts, including HNSCC (Figure S7C).

**Figure 6:**
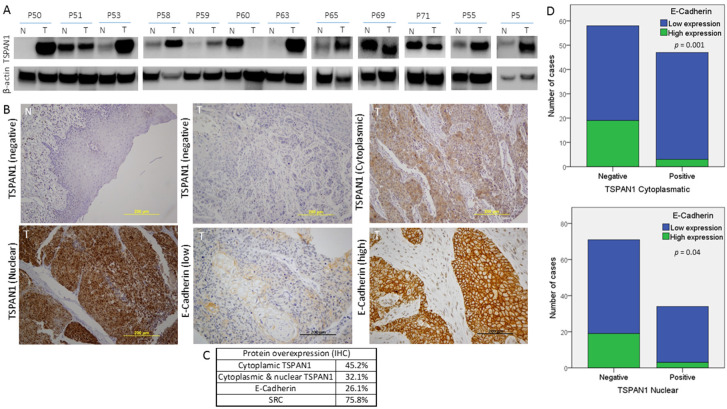
Protein expression study of TSPAN1 in human biopsies from HNSCC patients. (**A**) Western blot of TSPAN1 protein in laryngeal and pharyngeal cancer biopsies comparing tumor (T) vs. normal tissue (N). (**B**) Representative images of IHC staining for TSPAN1 and E-Cadherin proteins. The expression of TSPAN1 was only observed in the tumor tissue, showing either a cytoplasmic or a cytoplasmic and nuclear pattern. (**C**) Table summarizes the percentage of patients with positive expression for the indicated proteins in the studied cohort of 106 HNSCC patients. (**D**) Correlation between E-Cadherin and TSPAN1 expression. The plots show the expression of cytoplasmic TSPAN1 (upper panel) and nuclear TSPAN1 (lower panel). χ^2^ test and Fisher’s exact test.

**Figure 7:**
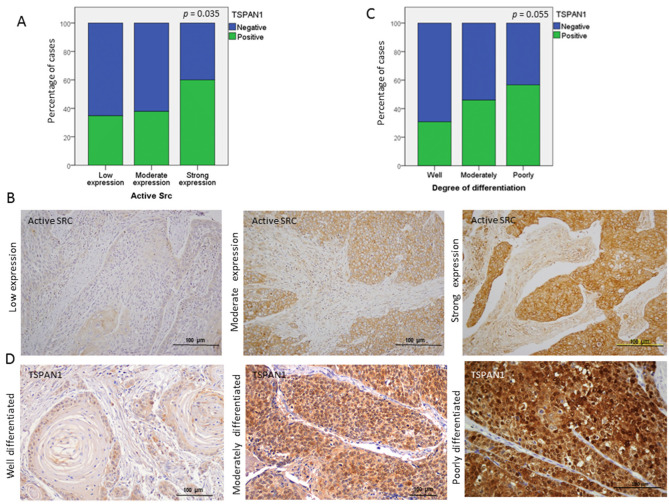
Associations of TSPAN1 expression with active SRC and with the degree of tumor differentiation. (**A**) Direct correlation of TSPAN1 expression with active SRC. χ^2^ test and Fisher’s exact test, * (*p* < 0.05). (**B**) Representative IHC images for the different expression levels of active SRC (low, moderate and high). (**C**) TSPAN1 expression tends to correlate with the degree of tumor differentiation, showing higher TSPAN1 expression in patients with poorly differentiated tumors. (**D**) Representative IHC images to illustrate TSPAN1 expression along different degrees of tumor differentiation (well, moderately and poorly) as indicated.

Secondly, TSPAN1 protein levels were analyzed by Western blot in paired samples of laryngeal and pharyngeal tumors (T) and patient-matched normal mucosa (N). TSPAN1 overexpression was detected in eight out of 12 tumor samples compared to the normal counterparts (Figure 6A and Figure S8). Of note, high TSPAN1 levels were also observed in some normal tissues comparable to the matched tumor. To further and significantly extend these data, immunohistochemical analysis of TSPAN1 were performed in a cohort of 106 laryngeal and pharyngeal cancer patients, and correlated with clinical data and disease outcome (Table S7). TSPAN1 protein expression in tumors exhibited cytoplasmic and nuclear patterns that were separately scored, whereas TSPAN1 expression was negligible in matched adjacent normal epithelia and stromal cells (Figure 6B). Although the cytoplasmic expression of TSPAN1 has been reported, we have not found previous works describing nuclear expression [26,31,33]. Forty-eight (45.2%) out of 106 patients showed cytoplasmic TSPAN1, and 34 of them showed concomitant cytoplasmic and nuclear staining (32.1% of total) (Figure 6C). Based on our data from HNSCC models, possible associations of TSPAN1 expression with p-SRC and E-cadherin were assessed. Interestingly, cytoplasmic and nuclear TSPAN1 were both inversely and significantly correlated with E-Cadherin expression (*p* = 0.001 and *p* = 0.04, respectively) (Figure 6D), confirming a possible link between TSPAN1 and EMT in HNSCC. In agreement with our results in HNSCC cells, TSPAN1 expression also correlated with active SRC (*p* = 0.035) (Figure 7A,B). In addition, we found a borderline association of TSPAN1 expression with a poor degree of histological differentiation of tumors (*p* = 0.055) (Figure 7C,D).

## 4. Discussion

Our results uncover TSPAN1 as a key protein in HNSCC pathogenesis, particularly in laryngeal and pharyngeal cancer. In the present study, a proteomic screen revealed that TSPAN1 protein is overexpressed in both CDDP-resistant cells and CSCs from CCL-138 cells. Regardless the endogenous level of TSPAN1 expression in different HNSCC cell lines (CCL-138, JHU029 or HTB-43), its inhibition by siRNAs, at RNA and protein level, consistently caused a decrease in proliferation and apoptosis induction. Moreover, the induction of apoptosis provoked by TSPAN1 inhibition was concomitantly accompanied by a decrease in autophagy. This result supports our previous findings indicating that autophagy activation is a feature associated with resistant phenotypes in HNSCC cells and tumors [20]. Accordingly, TSPAN1 inhibition has been linked to decreased cell proliferation and apoptosis induction, as previously reported in other cancer types. The upregulation of caspases 3 and 8, in conjunction with the downregulation of Bcl-2, are linked to apoptosis activation in these studies [34,35,36,37].

Importantly, TSPAN1 depletion was found to sensitize resistant cells to the action of distinct therapeutic drugs (i.e., CDDP and dasatinib), and this sensitization was more pronounced in biopsy-derived primary cell lines from laryngeal and pharyngeal tumors in comparison to established HNSCC cell lines. Although some reports have described several members of the tetraspanin family (i.e., CD9, CD81, TSPAN8, CD51) associated with CSC properties and/or resistance to current therapies in various cancer models [38,39,40,41,42]; as far as we know, this is the first time that TSPAN1 has been shown to be involved in the cellular response to chemotherapy.

Upon TSPAN1 inhibition, a decrease in p-SRC activation occurs as a common feature in all HNSCC cell lines tested, which was accompanied by a decrease in p-AKT and p-ERK1/2 levels. Nevertheless, TSPAN1 depletion decreased p-ERK1/2 in both parental JHU029 and resistant JHU029-R cells, while p-AKT levels only diminished in JHU029-R cells. Moreover, Vimentin was reduced in both parental JHU029 and resistant JHU029-R cells upon TSPAN1 depletion, suggestive of a link between TSPAN1 and the EMT process. However, vimentin reduction was not accompanied by an induction of E-Cadherin or a decrease in N-Cadherin expression. Therefore, our results suggest that SRC signaling is a major effector of TSPAN1 in these HNSCC models, with p-SRC emerging as a central node. However, the effects of TSPAN1 depletion extend beyond solely regulating p-SRC activation to also effectively target the levels of p-AKT, p-ERK1/2 and autophagy pathway. In marked contrast, the action of a SRC inhibitor such as dasatinib in JHU029-R cells was restricted to decrease p-SRC and p-ERK1/2 levels but did not affect p-AKT levels nor autophagy-related genes.

TSPAN1 has been described as a typical cell surface receptor that, like other tetraspanin family members, could be involved in the activation of various signaling cascades through interactions with other tetraspanins, integrins, receptors and/or cytoplasmic proteins (associations known as tetraspanin web). Therefore, TSPAN1 could activate additional effectors, apart from p-SRC, to contribute to the carcinogenesis process facilitating the acquisition of therapy resistance. In cholangiosarcoma and HNSCC models, the interaction of TSPAN1 with integrins has been described [31,43]. It would be worth to explore in the future if TSPAN1 activates p-SRC though the interaction with integrins or other cell surface receptors -including its own putative dimerization [28] and also its translocation from cytoplasmic to nuclear localization.

Interestingly, the biological effects of TSPAN1 depletion observed *in vitro*, were also corroborated in vivo. In the xenografted mice, we found that tumors formed by resistant JHU029-R cells were smaller in size but developed more metastasis than those formed by parental JHU029 cells [28]. Accordingly, all resistant HNSCC cell lines showed reduced proliferate capacity in culture than the corresponding parental cells. This can be due to the fact that resistant cells have a higher proportion of CSCs and autophagy upregulation, as we previously reported [20]. This supports the notion that resistant tumors (presumably enriched in number and features of CSCs and/or autophagy activation), are not necessarily bigger in size or more proliferative. In fact, they are instead more aggressive, a characteristic frequently observed in the clinic [44]. Specifically, CSC subpopulations are quite quiescent. Dynamic changes and complex interactions in the tumor microenvironment play an integral part during tumor progression and metastasis, as an adaptive response to enhance CSC survival [45]. In this line of evidence, tumors formed by slowly growing resistant cells JHU029-R showed a fusocellular pattern compared to parental JHU029 cells. Such pattern is associated with EMT features, which are typically prominent in aggressive and resistant tumor phenotypes [46]. Moreover, in tumors formed by resistant JHU029-R cells, TSPAN1 inhibition was able to revert the fusocellular pattern to an epithelial morphology and reduce metastasis capacity of these resistant HNSCC cells. These results confirm the oncogenic role of TSPAN1 in vivo in our laryngeal cancer model and suggest a putative role of TSPAN1 in EMT, supporting the observations described by Wang et al. in a cholangiocarcinoma model [31].

Importantly, the IHC and Western blot analysis of TSPAN1 in HNSCC patient biopsies, further confirm the potential oncogenic role of TSPAN1 in HNSCC pathogenesis. Thus, TSPAN1 expression was detected in ~45% (IHC) of laryngeal and pharyngeal tumors, a similar percentage to the upregulation of TSPAN1 detected at mRNA level (8 out of 16 patients) and also by Western blot (eight out of 12 patients). In good agreement, the overexpression of TSPAN1 in human cancer vs. adjacent non-cancerous tissue has been widely documented in cholangiocarcinoma [31,47,48], skin squamous cell carcinoma [36,49], esophageal carcinoma [34], ovarian carcinomas [50], prostate cancer [26], pancreatic cancer [35,37] and gastric carcinoma [51,52]. Moreover, TSPAN1 expression has been associated with HPV infection in cervical carcinomas, and considered an important diagnostic and prognostic marker, as strong TSPAN1 expression was found in a subset of high-grade cervical cancers and in most of the undifferentiated squamous cell carcinomas [53,54,55].

TSPAN1 protein showed predominantly a cytoplasmic and nuclear pattern inHNSCC specimens, whereas TSPAN1 expression was negligible in stromal cells and adjacent normal epithelia. Moreover, the IHC analysis on patient samples also corroborates the strong correlation between TSPAN1 and SRC activation previously observed in our HNSCC models. Similarly, IHC data also demonstrated an inverse correlation between cytoplasmic and nuclear TSPAN1 and E-Cadherin expression, thus reinforcing our in vitro findings suggesting a possible link between TSPAN1 and EMT. In addition, TSPAN1 expression was more frequently detected in poorly differentiated tumors (with higher EMT features) than in well differentiated (57% vs. 31%) (Figure 7). Together these results strongly support the involvement of TSPAN1 in EMT promotion in HNSCC.

Hence, all these observations lead us to propose for the first time TSPAN1 as an oncogenic protein involved in HNSCC chemoresistance, as well as in HNSCC development and progression, with p-SRC emerging as a predominant downstream effector. Moreover, TSPAN1 overexpression is associated to the development of an EMT program as consistently observed in mice tumors and HNSCC patient biopsies. Our findings support the notion that SRC activation may contribute to metastatic dissemination in HNSCC [25] and provide additional evidence extending the association of SRC activation with EMT to other HNSCC subtypes (i.e., laryngeal and pharyngeal carcinoma) beyond previous observations in nasopharyngeal carcinoma [56].

According to the herein presented data, we anticipate that TSPAN1 inhibition could represent a promising candidate for targeted therapy against laryngeal and pharyngeal cancers and possibly other HNSCC subsites. The fact that inhibition of TSPAN1 is able to sensitize resistant cells to different chemotherapeutic agents, has an important added value to be considered a potential novel target for cancer therapy.

## 5. Conclusions

Here, we described that TSPAN1 is a novel oncogenic protein associated with HNSCC chemoresistance, with SRC kinase signaling emerging as a central node. Mice tumors formed by cisplatin-resistant HNSCC cells showed a fusocellular pattern compared to parental cells. Importantly, TSPAN1 inhibition was able to revert this fusocellular pattern to an epithelial morphology, and also to reduce metastatic spreading. Consistent with these data, TSPAN1 expression in HNSCC patient biopsies was also correlated with EMT features and SRC activation.

## Data Availability

The mass spectrometry proteomics data (Tables S2–S5) have been deposited to the ProteomeXchange Consortium via the PRIDE partner repository [21] and are available with the dataset identifier PXD020159.

## Supplementary Materials

The following are available online at https://www.mdpi.com/2072-6694/12/11/3269/s1, Figure S1: TSPAN1 depletion inhibits cell proliferation, Figure S2: TSPAN1 inhibition under an alternative specific mix of 30 siRNA (siPool) sensitizes HNSCC cells to the effects of CDDP, Figure S3: TSPAN1 inhibition sensitizes HNSCC cells to the effects of dasatinib, Figure S4: TSPAN1 inhibition modulates different pathways. Figure S5: Phenotypic characteristics, TSPAN1 levels, and proliferative potential of tumors formed in mice. Figure S6: Ex vivo analysis of micrometastases under TSPAN1 depletion, Figure S7: TSPAN1 expression at mRNA level, Figure S8: Original western blot figures (uncropped blots), Table S1: General characteristics of the 106 patients analyzed in this study, Table S2. Proteins identified in the proteomic study. An ANOVA test was performed to determine proteins significantly dysregulated between CCL-138, CCL-138-R and CCL-138 CSCs, Table S3. ANOVA test to determine proteins significantly dysregulated between CCL-138, CCL-138-R and CCL-138 CSCs, Table S4. Post-Hoc analysis which shows the differences observed between CCL-138-R and CCL-138 cells, Table S5. Post-Hoc analysis which shows the differences observed between CCL-138 CSCs and CCL-138 cells, Table S6: Proteins commonly deregulated in CCL-138-R cells and CSCs versus CCL-138, Table S7: Clinical and pathological characteristics of the patients analyzed in this study.
